# Bilateral Stress Fractures of the Talus Associated with Adult-Acquired Flatfoot Deformities

**DOI:** 10.1155/2018/5376384

**Published:** 2018-09-09

**Authors:** Takumi Matsumoto, Song Ho Chang, Ryutaro Takeda, Sakae Tanaka, Takuo Juji

**Affiliations:** ^1^Department of Orthopaedic Surgery, Faculty of Medicine, The University of Tokyo, 7-3-1 Hongo, Bunkyo-ku, Tokyo 113-8655, Japan; ^2^Department of Rheumatology, JCHO Yugawara Hospital, 438 Miyakami, Yugawara, Ashigarashimo, Kanagawa 259-0396, Japan

## Abstract

Adult-acquired flatfoot deformity is a progressive flattening of the arch of the foot that results from posterior tibial tendon insufficiency with a predilection for middle-aged women. A lateralized force vector associated with hindfoot valgus in adult-acquired flatfoot produces lateral ankle pain due to impingement at the lateral hindfoot, which can even lead to stress fractures of the distal fibula. Here, we present the rare case of a 73-year-old woman who presented with stress fractures of the bilateral taluses and unilateral distal fibula accompanied by severe adult-acquired flatfoot deformities.

## 1. Introduction

Adult-acquired flatfoot deformity is a progressive flattening of the arch of the foot that results from a combination of posterior tibial tendon insufficiency and failure of the capsular and ligamentous structures of the foot. A variety of symptoms can accompany this deformity depending according to its progression including pain and swelling along the course of the posterior tibial tendon, a heavy or tired feeling in the foot, difficulty walking or standing, painful calluses on the sole, and pain in the lateral ankle [1]. Lateral hindfoot pain is generally due to impingement or arthrosis at the lateral hindfoot caused by an altered biomechanical force vector associated with hindfoot valgus [2]. Impingement on the distal fibula by the talus and/or calcaneus could even produce stress fracture of the distal fibula [3]; however, to our knowledge, no studies have reported stress fractures of the talus associated with adult-acquired flatfoot deformity. Here, we report an unusual case of bilateral stress fractures in the lateral talar body that occurred in an elderly female with a severe pes planovalgus deformity.

## 2. Case Report

A 73-year-old woman presented with the complaint of left ankle pain that had exacerbated with walking for the previous month. She had a limping gait and complained of a limited walking ability of 5–10 minutes with a cane because of pain. She originally had limited walking ability due to a cerebral infarction 10 years prior that was classified as least-limited community walker according to the classification by Perry et al. [4]; however, she had been able to walk without a cane for 30 minutes prior to symptom onset. An examination revealed bilateral hindfoot valgus and flatfoot deformities, and both feet were rigid and not reducible. The physical examination revealed localized swelling and tenderness on the distal fibula about 5 cm proximal to the tip of the left lateral malleolus.

**Figure 1:**
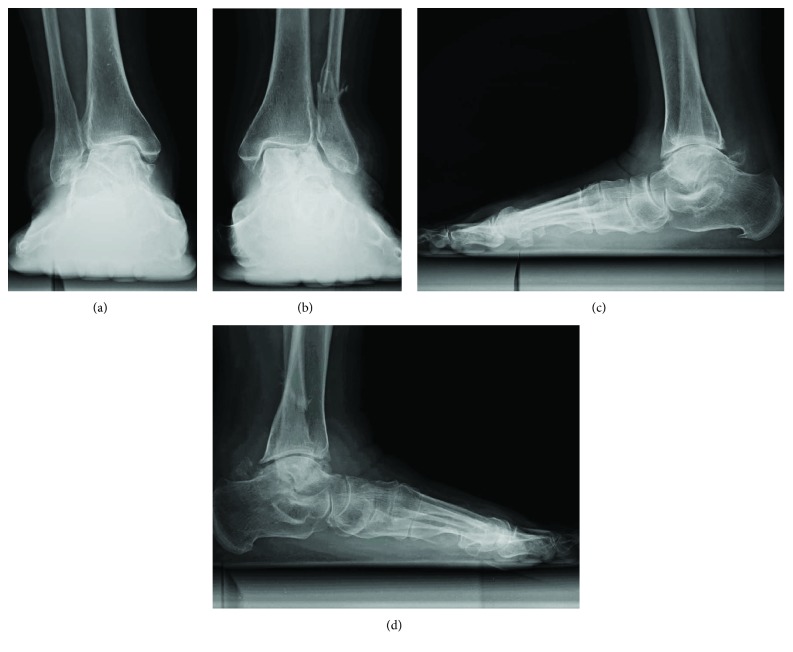
Plain radiographs obtained at the first visit. Anteroposterior weight-bearing radiograph of the right ankle (a) and left ankle (b) showing valgus talar tilt with joint space narrowing and collapse of the lateral talar dome. The left ankle was also accompanied by fracture of the distal fibula (b). Lateral weight-bearing view of the right foot (c) and left foot (d) showing the severe arch collapse and increased radiodensity of the body of the talus.

**Figure 2:**
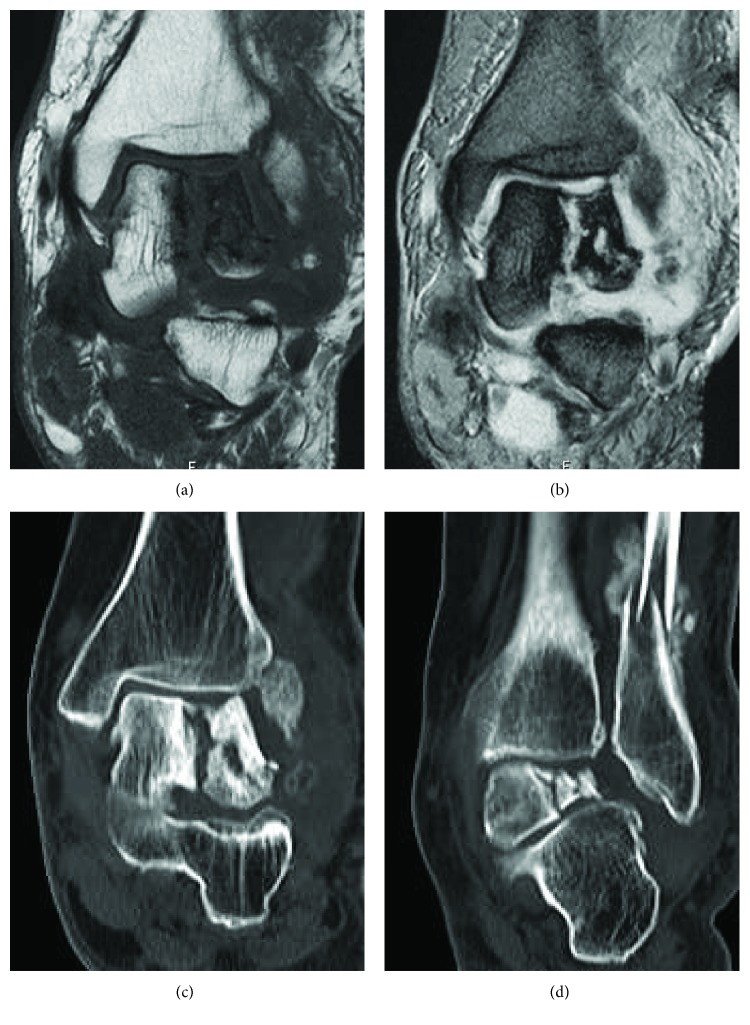
T1-weighted (a) and short-T1 inversion recovery (b) coronal magnetic resonance imaging of the left ankle showing a vertical fracture of the talar body and osteonecrosis of the lateral talar body fragment. Computed tomography images of the left ankle showing the comminuted fragments of the lateral talar body (c) and the fracture of the distal fibula with callus formation (d).

**Figure 3:**
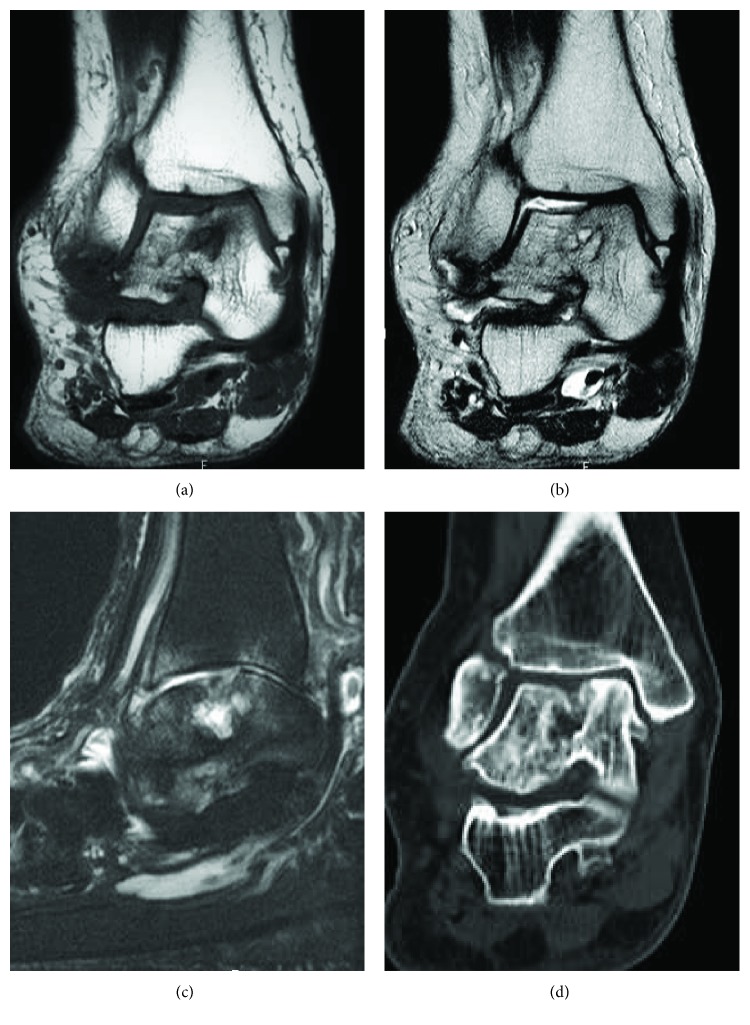
T1-weighted (a) and T2-weighted (b) coronal magnetic resonance images of the right ankle showing the depressed lateral talar dome and cyst formation in the talar body suggestive of a previous talar dome fracture. (c) T2-weighted spectral attenuated inversion recovery (SPAIR) image in the sagittal plane showing the cyst formation and surrounding bone marrow edema in the talus underneath the depressed lateral talar dome. (d) Computed tomography image of the right ankle suggesting a past fracture of the talar body and its bony union.

An anteroposterior (AP) weight-bearing view of the left ankle joint revealed the fracture of the distal fibula, valgus talar tilt with joint space narrowing at the lateral tibiotalar joint, and collapse of the lateral talar dome (Figure 1(b)). An AP weight-bearing view of the right ankle joint revealed similar findings on the contralateral side excluding the fracture of the distal fibula (Figure 1(a)). Lateral weight-bearing views of both feet demonstrated severe arch collapse and increased radiodensity of the body of the talus (Figures 1(c) and 1(d)). Magnetic resonance imaging (MRI) of the left ankle revealed a vertical crack in the talar body extending from the center of the talar dome to the subtalar joint and a lateral talar body fragment with low signal intensity on both T1-weighted and short T1 inversion recovery images suggestive of osteonecrosis (Figures 2(a) and 2(b)). A computed tomography (CT) scan of the left ankle clearly demonstrated that the fracture lines extended from the talar dome to the subtalar joint with the comminuted lateral talar body fragments and the fracture of the distal fibula with callus formation (Figures 2(c) and 2(d)). MRI of the right ankle also revealed a depressed lateral talar dome and some cyst formations surrounded by bone marrow edema in the talar body suggestive of a previous talar dome fracture; however, the lateral talar body fragment maintained the same intensity as those of other bone marrow on T1- and T2-weighted images, negating osteonecrosis in contrast with the left ankle (Figures 3(a)–3(c)). A CT scan of the right ankle demonstrated the continuity of the bone trabeculae in the suspected area of the fracture suggesting bony union (Figure 3(d)).

She could not recall any trauma or unusual activity that may have caused this problem. She denied any history of smoking, alcohol abuse, or corticosteroid use. A blood test was negative for rheumatoid factor, anticyclic citrullinated peptide antibody, and antinuclear antibodies. Radiographs of other joints including the hip, knee, and shoulder revealed no osteonecrosis. Dual-energy X-ray absorptiometry measurements showed that the bone mass was 82% of young adult mean (YAM) in the lumbar spine and 81% of the YAM in the femoral neck without any use of drugs affecting bone metabolism, which did not meet the criteria for osteoporosis.

The fibular stress fracture was treated successfully in 2 months with a walking boot and limited weight-bearing with crutches. After the fibular stress fracture healed, the patient could perform her activities of daily living as before the fibular fracture without wearing any devices or using any analgesics for pain control. At the time of the most recent follow-up, 2 years after the first visit to our hospital, the patient maintained the performance described above without complaint, although the comminuted lateral talar body displayed nonunion on radiography and CT evaluations.

## 3. Discussion

Stress fractures refer to fractures caused by mismatched bone strength and repetitive stress placed upon a bone. These fractures can be further divided into two types: fatigue fracture resulting from abnormal stresses on normal bone and insufficiency fracture resulting from normal stresses on abnormal bone. The foot and ankle are the most common locations of stress fracture, and the prevalence is highest at the tibia, calcaneus, and metatarsals, followed by the navicular, fibular, medial malleolus, and other tarsal bones [5, 6]. The talus is a relatively uncommon site of a stress fracture, and only a small number of case series have been reported since the first report by McGlone in 1965 [7]. Although most have been reported in a case series of fatigue stress fractures in athletes, military recruits, and sports enthusiasts [8–10], a few have been reported as insufficiency stress fractures in association with osteoporotic conditions like postmenopausal women and rheumatoid arthritis patients [11–13]. The talar head is the most common site of stress fractures through the talus, implying that repetitive compression of the talar head against the navicular during the push-off phase may play a major role in the pathology of stress fracture [7, 10, 13]. The present case demonstrated a sagittal shearing fracture according to Sneppen et al.'s talar fracture classification, which runs through the body of the talus from the trochlea to the subtalar joint [14]. Shearing fractures are rarely reported stress fractures in previous studies, including three cases in elite female gymnasts aged 15–17 years by Rossi and Dragoni and one case in a 32-year-old social soccer player by Kim et al. [15, 16]. Considering that the most common mechanism of injury resulting in a shearing-type traumatic talar body fracture is an axial loading injury to the talus [17], we considered that the repeated axial cyclic loads to the talus produced by pes planovalgus deformity played a role in the pathology of the rare fracture pattern in the present case.

Valgus hindfoot alignment has been demonstrated to result in the lateralization of the force vector in some cadaver studies [18, 19]. Friedman et al. demonstrated using an established cadaver model of the acquired flatfoot deformity that the flatfoot condition resulted in a significant lateral shift in the global contact area and the location of peak pressure on the talar dome [19]. Significant increases in both mean and peak pressure were also found [19]. In the clinical setting, lateralization of the force vector in the acquired flatfoot deformity was demonstrated to cause hindfoot impingement including talofibular, talocalcaneal, and calcaneofibular and subsequent stress fracture of the distal fibula [2, 3, 20]. These findings may explain the mechanism of stress fractures of the lateral talar body and distal fibula in the present case of late-stage adult-acquired flatfoot with irreducible hindfoot valgus.

When stress fracture is suspected, plain radiograph should be the first imaging modality considered because of its availability and low cost. However, stress fractures of the talus may be difficult to diagnose on radiographs especially in the early stages without displacement of the bone fragment [13]. The value of computed tomography is limited in diagnosing stress fracture in the early stage because of lower sensitivity and higher radiation exposure than other imaging modalities [21]. MRI has been demonstrated to be superior to scintigraphy in comparison of the sensitivity and specificity in diagnosing stress fracture; therefore, MRI may be considered next when plain radiograph is negative [22]. Because, when missed, stress fracture of the talus could progress to depression or avascular necrosis of the talus as demonstrated in the present case, we would like to emphasize the need of a high level of suspicion in order to accomplish timely diagnosis and management of this fracture.

The talus is predisposed to avascular necrosis, mainly after traumatic fractures due to its nature of a wide range of articular cartilage coverage around 60%, no muscular or tendinous attachments, and small nutrient vessels [23]. Although a lower likelihood than a traumatic fracture, a stress fracture of the talus is also associated with a risk of avascular necrosis [24]. The blood supply of the talar body is mostly dependent on the anastomotic artery in the tarsal canal, which has several main branches into the body expanding medially to posterolaterally [23]. Thus, the posterolateral aspect of the talus receiving the least blood supply is more likely to be affected by osteonecrosis than other lesions [25]. In the present case, one talus was affected by osteonecrosis of the fracture segment in the lateral talar body. Although our present case was satisfied with the present condition and did not require further intervention due to her low level of activities of daily living, careful follow-up should be conducted because a stress fracture or osteonecrosis of the talus could provoke new symptoms resulting from degenerative changes around the talus in the future [25, 26].

## 4. Conclusion

In conclusion, a stress fracture of the talus may be provoked in adult-acquired flatfoot deformity. The repetitive shearing force placed on the talus due to the lateralization of the load axis in the fixed hindfoot valgus is considered the pathology of this condition.
